# Supplementary material for: Utilizing Simulation Software to Develop Injection Molding Process Windows with High-Impact Polystyrene
Source: Polymers (Basel). 2025 Mar 8;17(6):718. doi: 10.3390/polym17060718 (PMC11944686; doi:10.3390/polym17060718)
Supplement: Supplementary file 1 [file polymers-17-00718-s001.zip › polymers-3483116-supplementary.pdf]

Supplementary Information: Additional Data Collected for the Development of Process Windows and Their Experimental Validation

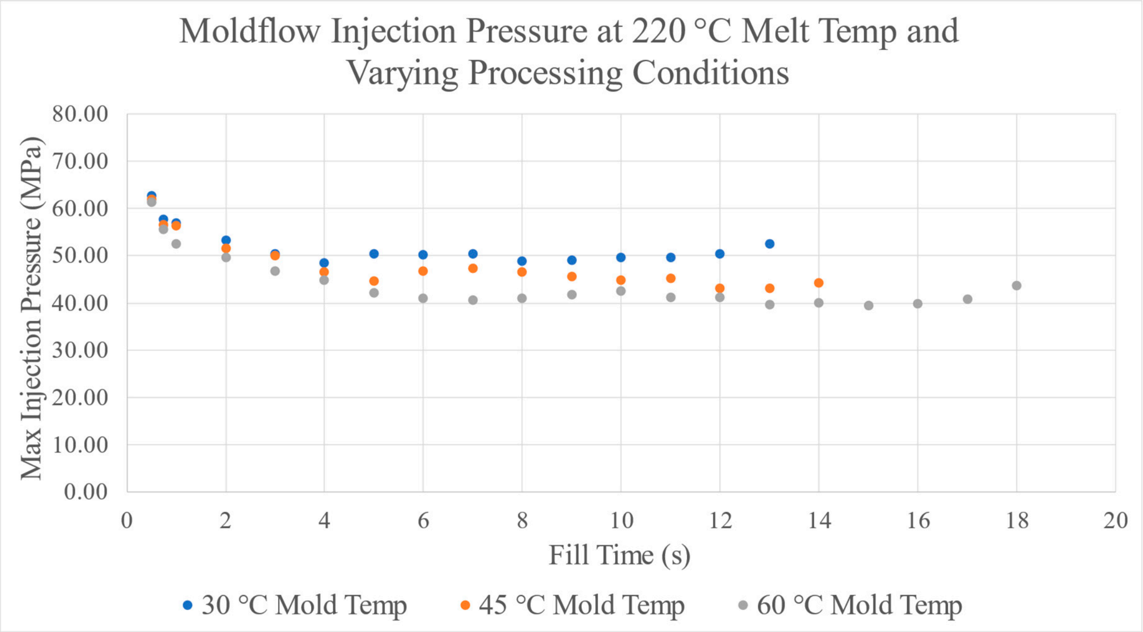

Figure S1: Moldflow Injection Pressures at 220 °C Melt Temp and Varying Process Conditions

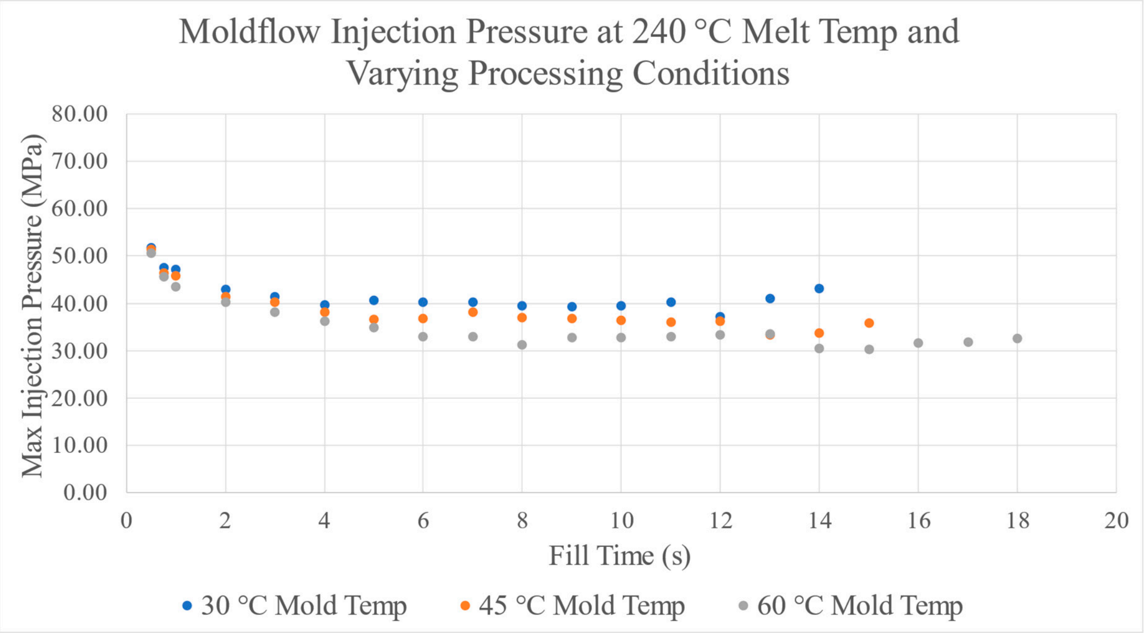

Figure S2: Moldflow Injection Pressures at 240 °C Melt Temp and Varying Process Conditions

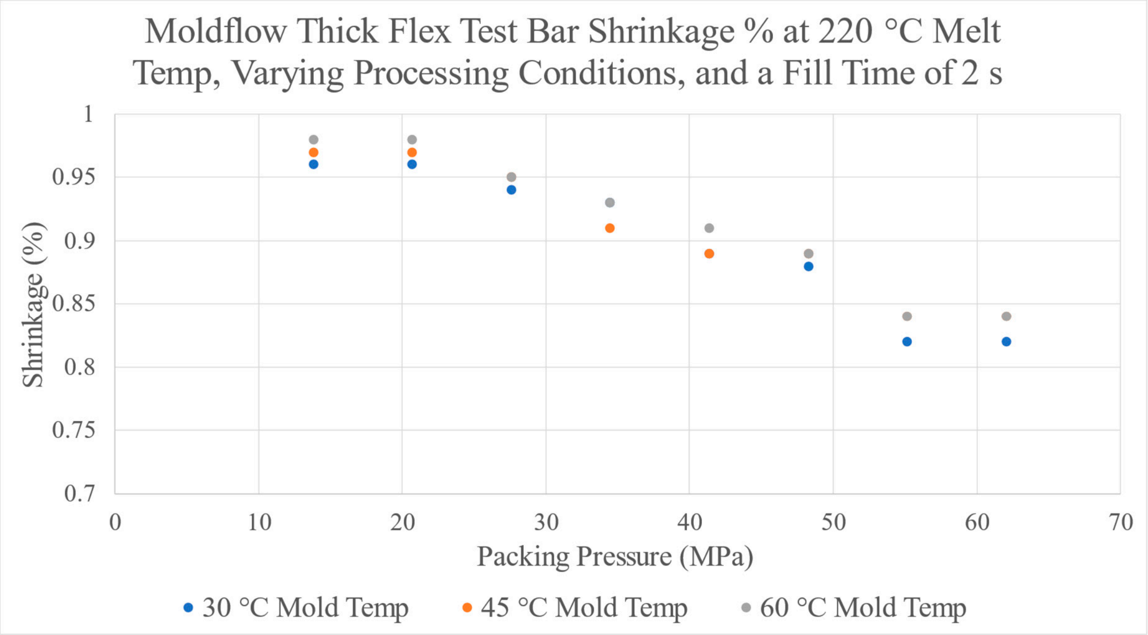

Figure S3: Moldflow Thick Flex Test Bar Shrinkage % at 220 °C Melt Temp and Varying Process Conditions

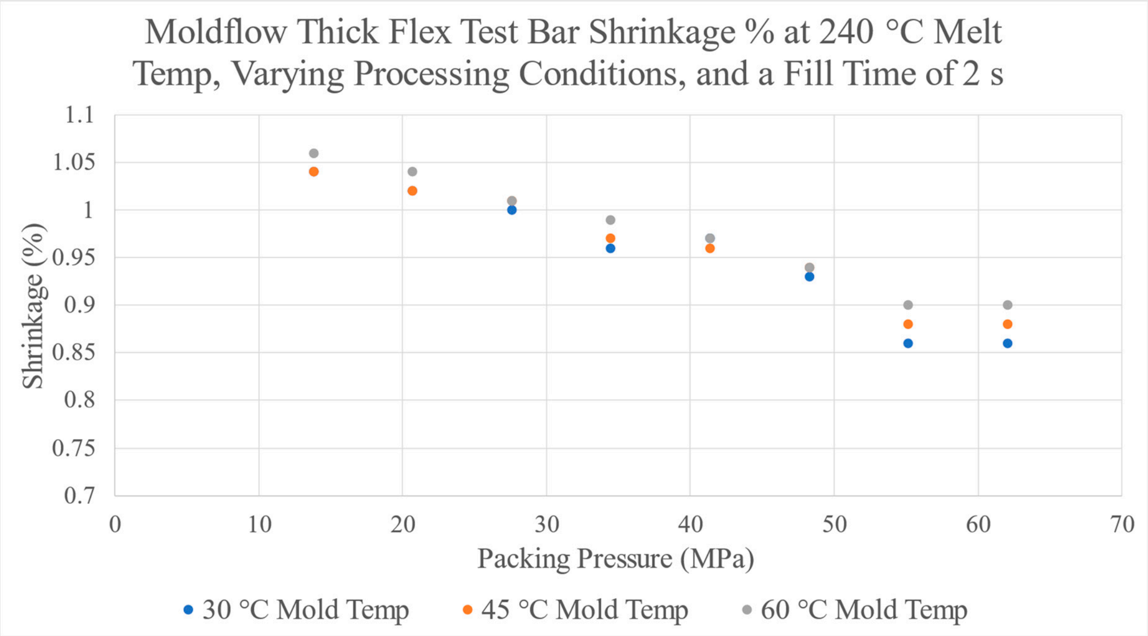

Figure S4: Moldflow Thick Flex Test Bar Shrinkage % at 220 °C Melt Temp and Varying Process Conditions
